# Knife ingestion in an eighteen months child

**DOI:** 10.11604/pamj.2020.36.336.25465

**Published:** 2020-08-25

**Authors:** Leila Debono, Nour Mekaoui

**Affiliations:** 1 Pediatric´s Medical Emergencies, Children's Hospital of Rabat, Rabat, Maroc

**Keywords:** Knife, ingestion, child, emergencies

## Image in medicine

A Male child of eighteen months old was admitted in the pediatric medical emergency department for ingestion of foreign body while he was playing with his seven years old sister. He was not presenting any symptoms. The clinical examination was normal: he was conscious, calm, with no respiratory distress. The inspection of oropharynx did not find any abrasions or lacerations and the abdomen examination was normal. A thoraco abdominal X-ray showed an image of a knife measuring 12cm between the lower third of the esophagus and the stomach. A second X-ray with the undressed child confirmed the diagnosis (A). A second anamnesis made the sister confess that they were watching a television show of sword swallowers when her brother ingested the knife. The patient was admitted in the operating room to extract the sharp object. The surgical removal did not find any lesions of the gastrointestinal tract and was successful (B).

**Figure 1:**
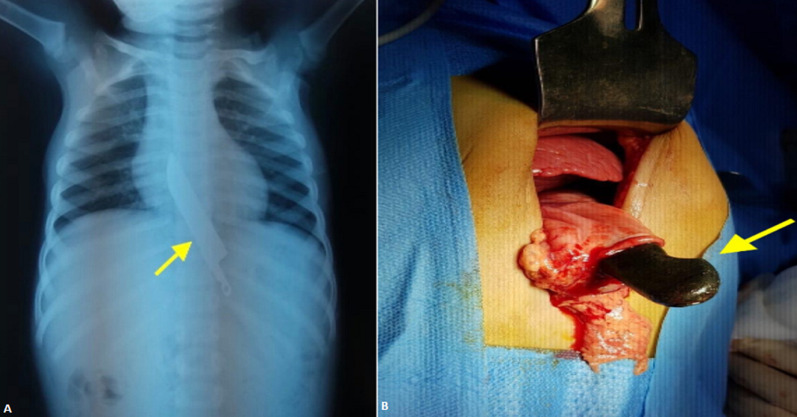
knife ingestion in an eighteen months child (A,B)

